# High efficacy of two artemisinin-based combinations: artesunate + sulfadoxine-pyrimethamine and artemether-lumefantrine for falciparum malaria in Yemen

**DOI:** 10.1186/s12936-015-0970-2

**Published:** 2015-11-14

**Authors:** Ahmed A. Adeel, Niaz Abdo Saeed, Adel Aljasari, Amar M. Almohager, Mohamed H. Galab, Amar AlMahdi, Mansor H. Mahammed, Mohammed AlDarsi, Yahiya A. Salaeah, Hoda Atta, Ghasem Zamani, Marian Warsame, Amy Barrette, Hanan El Mohammady, Rania A. Nada

**Affiliations:** College of Medicine, King Saud University, Riyadh, Saudi Arabia; The National Malaria Control Programme, Ministry of Public Health and Population, Sanaa, Yemen; Ministry of Public Health and Population, Sanaa, Yemen; Malaria Control and Elimination, Division of Communicable Diseases Control, World Health Organization Regional Office for the Eastern Mediterranean, Cairo, Egypt; Global Malaria Programme, World Health Organization, Geneva, Switzerland; Naval Medical Research Unit-3, Cairo, Egypt

**Keywords:** Yemen, Artesunate, Sulfadoxine-pyrimethamine, Anti-malarial drugs, Drug resistance, *Plasmodium falciparum*, Gametocytaemia, Artemether-lumefantrine

## Abstract

**Background:**

Artesunate + sulfadoxine-pyrimethamine (AS + SP) has been the first-line treatment and artemether-lumefantrine (AL) the second-line treatment for uncomplicated falciparum malaria in Yemen since 2005. This paper reports the results of studies conducted to monitor therapeutic efficacy of these two drugs in sentinel sites in Yemen.

**Methods:**

Eight therapeutic efficacy studies were conducted in six sentinel sites during the period 2009–2013 in Yemen. Five studies were for the evaluation of AS + SP (total of 465 patients) and three studies (total of 268 patients) for the evaluation of AL. The studies were done according to standard WHO protocol 2009 with 28-day follow-up.

**Results:**

In the evaluation of AS + SP, the PCR-corrected cure rate was 98 % (95 % CI 92.2–99.5 %) in one site and 100 % in all of the other four sites. In the sites where AL was evaluated, the PCR-corrected cure rate was 100 % in all the sites. All patients were negative for asexual parasitaemia on day 3 in both the AS + SP and the AL groups. There was a higher rate of clearance of gametocytaemia in the AL-treated group when compared with the AS + SP groups from day 7 onwards.

**Conclusion:**

AS + SP remains the effective drug for uncomplicated falciparum malaria in Yemen. AL is also highly effective and can be an appropriate alternative to AS + SP for the treatment of falciparum malaria. AL demonstrated a higher efficacy in clearing microscopic gametocytaemia than AS + SP.

Trial registration: Trial registration number ACTRN12610000696099

## Background

There are four major epidemiological strata of malaria in the Republic of Yemen. These are coastal plain, foothills, mountains, and the island of Socotra. Malaria-free zones include the mountain plateau and arid slopes from the highlands to the desert. In 2013, 25 % (6,100,000) of a total population of 24,400,000 lived in areas of high transmission (>1 case/1000), 53 % (12,900,000) in areas of low transmission (0–1 case/1000) and 22 % (5,400,000) in malaria-free areas [1]. Most of the cases (99 %) are due to *Plasmodium falciparum* and only 1 % due to *Plasmodium vivax*. No indigenous malaria has been detected in Socotra since 2006 [2]. Malaria transmission in Yemen differs between the regions. In the coastal areas, peak transmission occurs in winter (October–April), while in the mountainous hinterland areas it usually peaks in summer (May–September). However, in highland areas located above 2000 m above sea level, transmission occurs throughout the year [3]. The geographic location of Yemen makes it highly relevant to the malaria elimination efforts of its northern neighbour (Saudi Arabia) and for the prevention of re-introduction of malaria of its eastern neighbour (Oman). Given that Yemen contributes over 98 % of the malaria burden of the Arab Peninsula, the achievement of the targets of ‘malaria Free Arabian Peninsula Initiative’ depends heavily on the disease situation in Yemen [2].

Most of the early studies on anti-malarial drug efficacy that were carried out in Yemen in the 1980s and the 1990s were done in the southern parts of the country. These were mainly in vivo studies based on the standard WHO seven-day test to assess response of falciparum malaria to chloroquine (CQ). The studies, which were conducted by WHO consultants for malaria control, reported no significant levels of CQ resistance at the time [4]. In 2002, the National Malaria Control Programme (NMCP) established sentinel sites for monitoring the therapeutic efficacy of anti-malarial drugs to *P. falciparum* based on the earlier versions of standard WHO protocol [5]. Since then, 17 studies have been conducted by the NMCP, covering CQ, sulfadoxine-pyrimethamine (SP) and amodiaquine (AQ) monotherapy and more recently, the artemisinin-based combinations of artesunate + amodiaquine (AS + AQ), artesunate + sulfadoxine-pyrimethamine (AS + SP) and artemether + lumefantrine (AL) (Adel Aljasary, pers. comm.). The first therapeutic tests on CQ were conducted in 2002–03 [6, 7] based on the 2001 WHO protocol [5]. By 2004, these tests revealed high rates of treatment failures with CQ and the results were summarized in WHO’s report on global monitoring of susceptibility of *Plasmodium falciparum* to anti-malarial drugs, which cited nine therapeutic efficacy studies in Yemen with median treatment failure rate of 42.4 % (range 9–57 %) for CQ and one trial for SP with no treatment failure [8].

The emergence and spread of drug-resistant malaria has been a major factor in the global resurgence of falciparum malaria in the 20th Century. Drug resistance has been implicated in the spread of malaria to new areas and re-emergence of malaria in areas where the disease had been eliminated. Drug resistance has also played a significant role in the occurrence and severity of epidemics in some parts of the world [9]. In Yemen, the spread of CQ-resistant falciparum malaria was associated with deterioration of the malaria situation in terms of disease prevalence and clinical impact [10]. The experience of Yemen with CQ resistance brought attention to the significance of surveillance of the efficacy of anti-malarial drugs as a main activity of the NMCP.

In 2005, Yemen switched to artemisinin-based combination therapy (ACT) for the treatment of uncomplicated falciparum malaria as per the recommendation of WHO [11]. The selection of the exact ACT is based on consideration of the options and the local resistance patterns for the partner drug [12, 13]. Efficacy trials done by the NMCP in Ibb in 2004 found that treatment failure rate after AQ monotherapy was 44 % and with AS + AQ 18.5 % (Adel Aljasary, pers. comm.). AS + AQ was excluded as an option in Yemen and led to selection of AS + SP as the first-line drug and AL as the second-line drug for uncomplicated falciparum malaria (Adel Aljasary, pers. comm.).

AS + SP has been evaluated extensively in adults and children with uncomplicated malaria in other parts of the world and was found to be sufficiently efficacious in areas where 28-day cure rates with SP alone exceeded 80 % [13, 14]. AL is currently one of the most widely used ACT for treatment of uncomplicated falciparum malaria. The previous four-dose regime was associated with 15 % treatment failure, but the now-recommended, six-dose regime has shown higher efficacy [15]. This paper presents the results of studies conducted to assess therapeutic efficacy of AS + SP and AL against falciparum malaria in Yemen from 2009 to 2013. In addition, the impact of these two ACTs on microscopic gametocytaemia were evaluated in the study population. These studies were carried in the framework of monitoring therapeutic efficacy of anti-malarial drugs.

## Methods

### Study design and patients

**Fig. 1:**
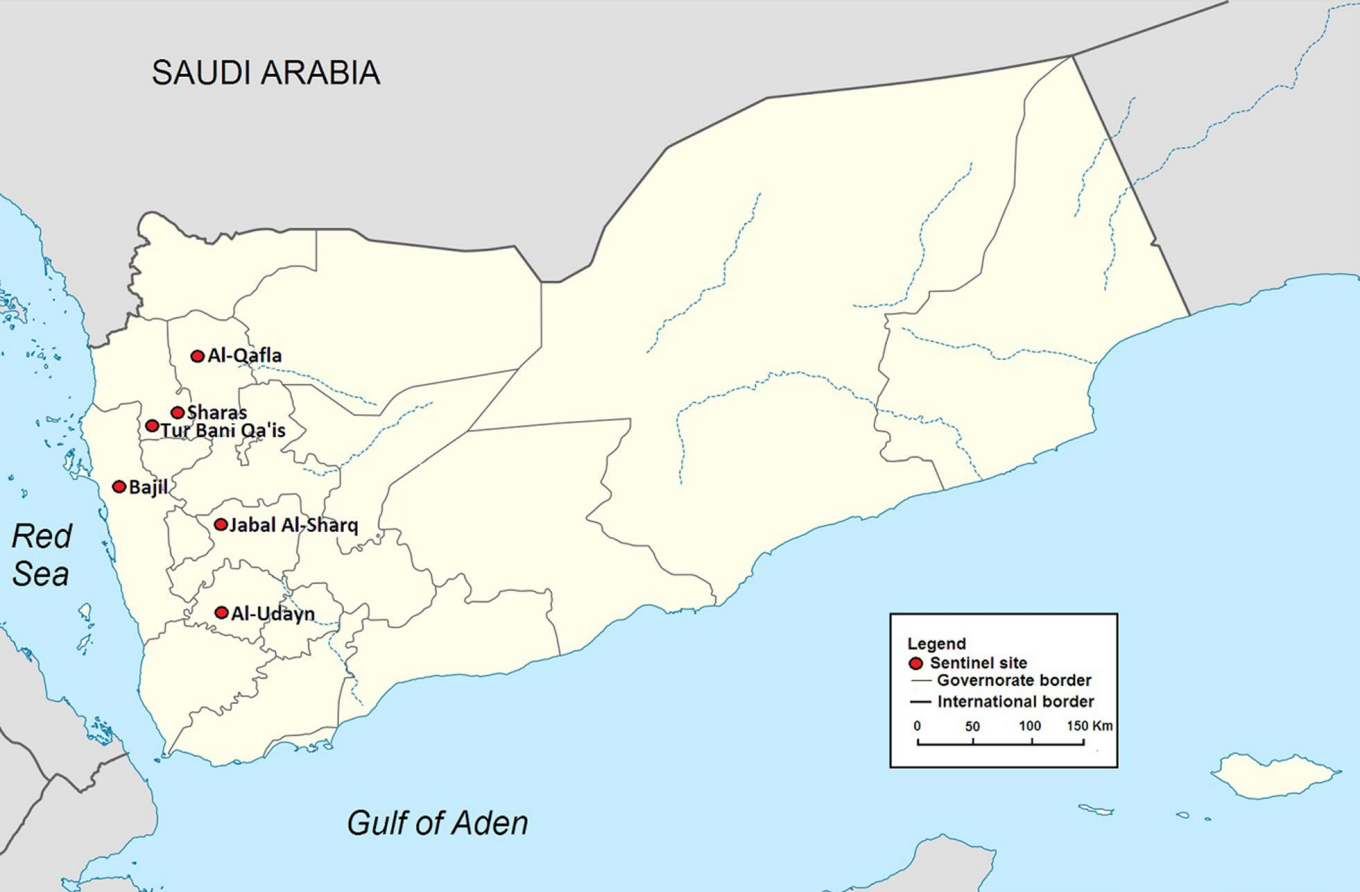
Map of Yemen showing the sites of therapeutic efficacy studies of anti-malarial drugs against falciparum malaria in Yemen 2009–2013

The studies were conducted in six sentinel sites representing the different malaria-endemic provinces of the country (see Fig. 1). Each study was timed to coincide with the peak of the malaria transmission in the area. The design of the study was an open-label, one-arm, prospective evaluation of clinical and parasitological responses to directly observed treatment for uncomplicated malaria. The WHO protocol for assessing therapeutic efficacy [16] was used. Cases were enrolled in the study, after obtaining the patients’ or parents’/guardians’ written consent, if they were aged 6 months and above, had symptoms compatible with uncomplicated clinical malaria with fever (body temperature ≥37.5 °C) or history of fever in the previous 24 h and mono-infection of *P. falciparum* with parasite density of 500–200,000 asexual parasites/μl. A patient was excluded if she/he had a febrile illness other than malaria or severe and complicated falciparum malaria.

Pregnant women and females of reproductive age who could not be tested for pregnancy were excluded. Pregnancy testing of unmarried women and female minors aged 12–17 years in Yemen is not acceptable according to the local customs and culture.

### Treatment and laboratory analysis

The methods of treatment, follow-up and analysis of outcomes was based on WHO guidelines [16]. After obtaining a written informed consent, a complete medical history was obtained and a complete physical examination was performed. Blood was collected for microscopy and on filter paper on day 0 before treatment and on days 2, 3, 7, 14, 21, 28 or on any other day if the patient returned spontaneously.

### Treatment regimen

All patients received a standard regimen of the drug being tested [12]. In the AS + SP studies the patients received a dose of 4 mg/kg/day artesunate once a day for 3 days and a single administration of 25/1.25 mg/kg SP on day 0, with a therapeutic dose range between 2 and 10 mg/kg/day artesunate and 25–70/1.25–3.5 mg/kg SP. In studies on AL, the patients were given AL (Coartem^®^, Novartis Pharmaceutical Corporation) according to body weight bands. Patients weighing 5–14 kg received one tablet (20 mg artemether plus 120 mg lumefantrine) per dose, those weighing 15–24 kg received two, those weighing 25–34 kg three tablets, and those weighing ≥35 kg received four tablets. In total, six doses were administered at hours 0, 8, 24, 36, 48, and 60. Study medications were provided by WHO. The intake of all doses of treatment was directly observed. All drugs were within their expiry period, and batch number and expiry date were recorded in each case record form (CRF). If a patient vomited within 30 min of treatment, a full dose was re-administered. All patients were followed up on days 1, 2, 3, 7, 14, 21, and 28. On each of these follow-up visits clinical and parasitological assessments were repeated. Patients were also asked to report to the clinic at any time if new or recurrent symptoms occurred.

### Rescue medication

All patients who failed to be cured with AS + SP were treated with AL. Those who failed treatment with AL were treated with quinine orally at 10 mg salt/kg three times daily for 7 days.

### Malaria microscopy

Thick and thin blood films for parasite counts were obtained from each patient and examined at screening and on days 2, 3, 7, 14, 21, 28 or on any other day if the patient returned spontaneously and parasitological reassessment was required. Blood smears were stained with 2.5 % Giemsa for 45 min and examined at a magnification of 1000× to identify the parasite species and to determine the parasite density.

The thick blood smear was used to calculate the parasite density, by counting the number of asexual parasites against 200 white blood cells (WBC) with a hand tally counter. The count was terminated when either 500 parasites or 200 WBC were reached, whichever came first. A blood smear was declared negative after examination of 1000 WBC revealed no asexual parasites. Parasite density, expressed as the number of asexual parasites per µl of blood, was calculated by dividing the number of asexual parasites by the number of WBC counted and then multiplying by an assumed WBC density of 8000 per µl.

For quality assurance, all blood smears were re-read by a second microscopist. Blood smears with non-concordant results (differences in species or differences in parasite density of >50 %) were re-read by a third microscopist and the average parasite density of the two most concordant counts was used.

### Genotyping of malaria parasites

Two to three drops of blood were collected through finger pricks on filter paper (Whatmann No 3) during enrolment and each time blood smears were required, according to the protocol, on and after day 7. Specimens were labelled anonymously (Patient ID number, day of follow-up, date), kept in individual plastic bags with desiccant pouches and protected from light, humidity and extreme temperature until analysed. The specimens were genotyped to distinguish between recrudescence and new infections, according to methods recommended by WHO [17]. Blood spots were tested using nested polymerase chain reaction (PCR) targeting polymorphic variant genes *msp1*, *msp2* (merozoite surface proteins) and *glurp* (glutamate-rich protein). Subsequently, gel analysis was performed using Bionumerics V.5.10 in order to determine the size of the amplified gene targets. Identification of recrudescence and new infections was performed according to WHO guidelines [17].

### Classification of treatment outcome

Treatment outcomes were classified on the basis of an assessment of the parasitological and clinical outcome of anti-malarial treatment according to WHO guidelines [16]. A patient was classified as having either early treatment failure (ETF), late clinical failure (LCF), late parasitological failure (LPF), or an adequate clinical and parasitological response (ACPR), as defined in guidelines [16]. Patients who were lost to follow-up, had re-infections or unknown PCR were excluded from the per-protocol analysis of treatment outcomes, but included in the Kaplan–Meier analysis until the day of withdrawal from the study.

### Drug tolerability and safety

Patients were assessed clinically for drug tolerability. Both adverse events and serious adverse events were monitored at enrolment and on each of the follow-up visit. An adverse event was defined as any untoward medical occurrence irrespective of its suspected relationship to the study medications. Serious adverse event included untoward medical occurrence requiring hospitalization or resulting in death.

### Sample size and statistical analysis

The treatment failure rate of the two drugs in the study areas was estimated to be 5 %. At a confidence level of 95 % and a precision estimate of 5 %, a minimum of 73 patients had been planned to be enrolled in each site. With a 20 % increase to allow loss to follow-up and withdrawals during the 28-day follow-up period, 87 patients were targeted to be included in the study per site per drug. Data were double entered and validated using a programme developed by WHO [16]. Per protocol and Kaplan–Meier survival analysis were used to evaluate the treatment outcome and only patients who could be evaluated on the respective days of follow-up were included in the analysis. The proportion of positive blood films on day 3 was recorded. Geometrical means of parasite density on day 0 were calculated for each site.

Statistical analysis was performed using SPSS 22^®^ to generate descriptive statistics and analyse data (SPSS Inc. Chicago, USA). A 2 × 2 Chi square table was used to analyse associations between proportions using Epi-Info 7 (Centers for Disease Control and Prevention, Atlanta, GA, USA and WHO, Geneva, Switzerland).

### Ethical considerations

Permission to conduct the studies was obtained from the General Doctorate for Research and Studies, Ministry of Health, Yemen, which is the national body with oversight to ethically review research proposals involving human subjects, and WHO Ethical Research Committee. Local authorities (community leaders) of the study areas were informed of the study objectives, procedures and duration and their permission was sought. Individual informed consent was obtained from adults and parents/guardians of children. Those who were illiterate selected a witness not related to the research team to sign on their behalf.

## Results

Eight therapeutic efficacy studies were conducted in six sentinel sites in Yemen to assess the therapeutic efficacy of AS + SP (five studies) and AL (three studies) during the period November 2009 to March 2013. Table 1 shows the demographic and clinical characteristics in the study population in each site. The age of patients ranged between 6 months and 75 years. The percentage of children under 5 years of age varied by study, ranging between 8.6 % in Tur Bani Qa’ is 2013 to 56 % in al-Udayn 2010. There were also differences in gender composition in the different studies, the percentage of males ranged between 46.7 and 72 %. The mean temperature at baseline showed variation ranging between 37.6 and 38.0 °C and the geometric mean asexual parasitaemia/µl varied from 1729 to 14,543/µl.

**Table 1:** Patient characteristics on admission, Yemen (2010–2013)

Drug	Site	Year	n	Males	(%)	Age <5 years		Temp °C	Febrile patients^a^ ≥37.5 n (%)	Parasitaemia (/μL)
						n (%)	95 % CI	Mean (SD)		Geometric mean (95 % CI)
AS + SP	Al-Udayn	2010	85	44	(51.8)	48 (56.5)	(45.3–67.2)	37.9 (0.2)	70 (82.4)	3429 (2643–4449)
AS + SP	Sharas	2010	93	52	(55.9)	31 (33.3)	(23.9–43.9)	37.7 (0.1)	56 (60.2)	9088 (6947–11,890)
AS + SP	Tur Bani Qa’is	2010	95	58	(61.1)	38 (40)	(30.1–50.6)	37.9 (0.3)	75 (78.9)	14543 (10,325–18,628)
AS + SP	Al Qaflaha	2011	90	42	(46.7)	33 (36.7)	(26.7–47.5)	37.6 (0.7)	55 (61.1)	3238 (2506–4184)
AS + SP	Bajil	2013	102	51	(50.0)	10 (9.8)	(4.8–17.3)	38.0 (0.4)	45 (44.1)	7011 (5461–8999)
AL	Bajil	2009–10	80	43	(53.8)	7 (8.8)	(3.6–17.2	37.6 (0.4)	74 (92.5)	12,606 (8707–18,248)
AL	Jabal Al Sharq	2010	95	57	(60.0)	14 (14.7)	(8.3–23.5)	37.6 (0.3)	57 (60)	1729 (1399–2137)
AL	Tur Bani Qa’is	2013	93	67	(72.0)	8 (8.6)	(3.8–16.2)	38.0 (0.1)	81 (87.1)	11,872 (8698–16,203)

^a^Febrile patients: axillary temp ≥ 37.5 °C

Table 2 shows details of the outcomes of all the studies. In the AS + SP studies, a total of 465 patients with falciparum malaria who satisfied the inclusion criteria were enrolled. The study was completed by 432 patients, with 426 cases classified on day 28 as ACPR. There were three LCF and two LPF. In addition, there were ten patients lost to follow-up and 24 withdrawals. The reasons for withdawal were quality control detecting that parasite count at baseline was too low (18 cases), mixed infection (two cases), underdose (one case), and withdrawal of consent by patient (three cases). According to the follow-up and analysis plan, cases initially classified as LCF or LPF were checked by PCR to differentiate recrudescence from re-infection. PCR-correction confirmed recrudescence in two of the LCF cases.

**Table 2:** Parasitological and clinical outcomes among patients treated with artesunate + sulfadoxine-pyrimethamine and artemether-lumefantrine after 28 days of follow-up, Yemen (2010–2013)

Drug	Site	Year	n	PD3+	Without PCR-correction						With PCR-correction					
					Excluded/loss	LCF	LPF	ACPR	Cure rate (KM)		Excluded/loss	LCF	LPF	ACPR	Cure rate (KM)	
				n (%)	n (%)	n (%)	n (%)	n (%)	%	(95 % CI)	n (%)	n (%)	n (%)	n (%)	(%)	(95 % CI)
AS + SP	Al-Udayn	2010	85	0 (0)	2 (2.4)	0 (0)	0 (0)	83 (100)	100	(95.7–100)	2 (2.4)	0 (0)	0 (0)	83 (100)	100	(95.7–100)
AS + SP	Sharas	2010	93	0 (0)	11 (11.8)	0 (0)	0 (0)	82 (100)	100	(95.6–100)	11 (11.8)	0 (0)	0 (0)	82 (100)	100	(95.6–100)
AS + SP	Tur Bani Qa’is	2010	95	0 (0)	12 (13.7)	1 (1.2)	1(1.2)	81 (97.6)	97.6	(91.8–99.4)	14(14.7)	0 (0)	0 (0)	81 (100)	100	(95.9–100)
AS + SP	Al-Qaflah	2011	90	0 (0)	6 (6.7)	0 (0)	0 (0)	84 (100)	100	(95.7–100)	6 (6.7)	0 (0)	0 (0)	84 (100)	100	(95.7–100)
AS + SP	Bajil	2013	102	0 (0)	3 (2.9)	1 (1)	2 (2)	96 (97)	96	(89.6–98.5)	4 (3.9)	2 (2)	0 (0)	96 (98)	98	(92.2–99.5)
AL	Bajil	2009	80	0 (0)	6 (7.5)	0 (0)	0 (0)	74 (100)	100	(95.1–100)	6 (7.5)	0 (0)	0 (0)	74 (100)	100	(95.1–100)
AL	Jabal-Al-Sharq	2010	95	1 (1.1)	5 (5.3)	0 (0)	2 (2.2)	88 (97.8)	97.8	(92.2–99.7)	7 (7.4)	0 (0)	0 (0)	88 (100)	100	(95.9–100)
AL	Tur Bani Qa’is	2013	93	0 (0)	5 (5.4)	5 (5.7)	0 (0)	83 (94.3)	94.3	(87.2–98.1)	10 (10.8)	0 (0)	0 (0)	83 (100)	100	(95.7–100)

*LCF* late clinical failure, *LPF* late parasitological failure, *ACPR* adequate clinical and parasitological response, *KM* Kaplan–Meier, *CI* confidence interval, *PD3*+ positive blood film on day 3

In the individual studies and after PCR-correction Kaplan–Meier analysis, the cure rate with AS + SP ranged between 98 and 100 % in the different sites. The two cases of treatment failure were in Bajil 2013. The first case (LCF) was a 2 year old male who presented on day 28 with a history of fever, his blood film showed 880 *P. falciparum* asexual stages/µl. The second case (LCF) was a 5 year old female who presented on day 28 with a recent history of fever and her blood film was positive with 45,454 *P. falciparum* asexual stages/µl. Both cases had treatment doses within the therapeutic range.

In the AL studies, a total of 268 patients with falciparum malaria satisfied the inclusion criteria (Table 1). The studies were completed by 252 patients with 245 cases classified on day 28 as ACPR, two cases as LPF in Jabal-Al-Sharq 2010, and five cases in Tor-Bani-Qa’is as LCF. In addition, there were six patients lost to follow-up and ten withdrawals. The reasons for withdrawal was quality control detecting low baseline parasitaemia (seven cases) and withdrawal of consent (three cases). According to the follow-up and analysis plan, cases initially classified as LCF or LPF were checked by PCR for recrudescence or re-infection, which found the five cases initially classified at LCF in Tur-Bani-Qa’is to be re-infections; the two cases initially classified as LPF (from Jabal-Al-sharq) were excluded from the PCR-corrected analysis because they had unknown PCR. In the PCR-corrected analysis and after Kaplan–Meier analysis, the cure rate following AL was 100 % in all the three sites tested. All cases except one had cleared asexual parasitaemia by day 3. Details of the PCR-uncorrected treatment failure cases were as follows: the first one was a 4 year-old female, who presented with history of fever and with 1400 asexual stages/µl on day 0. Low parasite count persisted on day 2 (520 asexual stages/µl), day 3 (320 asexual stages/µl) and day 7, when the parasite count went up to 5502 asexual stages/µl. She was classified as LPF, and because her PCR was negative on day 7 she was excluded in the PCR-corrected classification. This was the only case that had microscopic parasitaemia on day 3. The second case was a 7 year old, who presented with history of fever, parasite density on day 0 was 1200 asexual stages/µl, and had adequate resolution of fever and microscopic parasitaemia but on day 21 the parasite count was 7000 asexual stage/µl. The patient was classified as LPF but excluded from the PCR classification because the PCR was not known. The five cases of LCF in Tur-Bani-Qa’is were all children between 6 and 10 years old, who presented with fever and positive blood film on day 21 (one case) and day 28 (four cases). PCR analysis classified all five cases as re-infections. All therapeutic regimens of the two study drugs were well tolerated and safe.

**Fig. 2:**
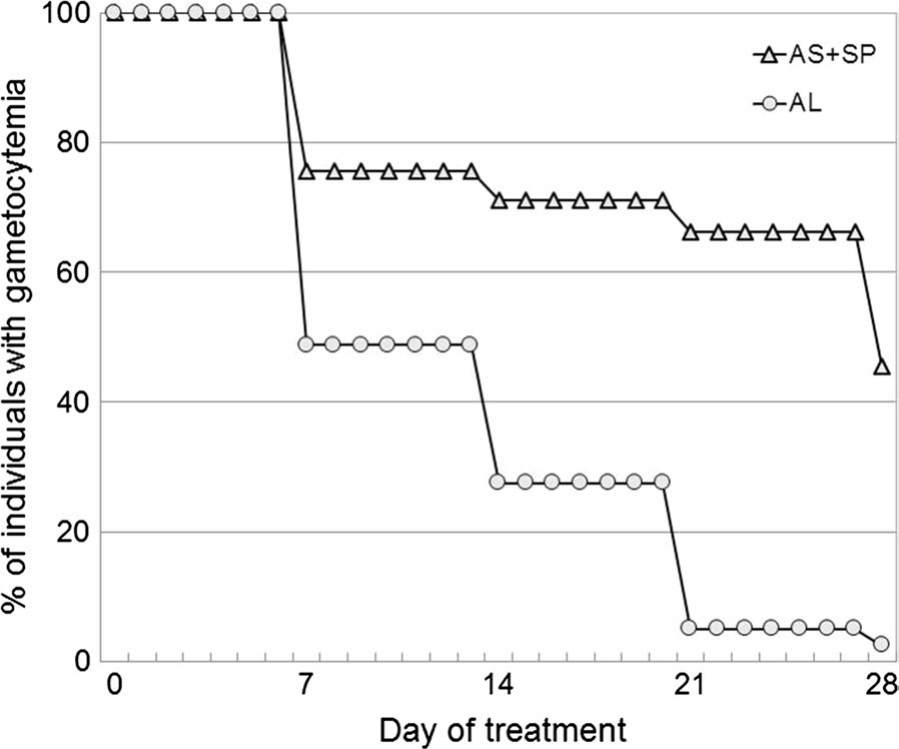
Time to disappearance of microscopic gametocytaemia in gametocyte-positive individuals at enrolment and following treatment. *AL* artemether-lumefantrine (n = 42), *AS* *+* *SP* sulfadoxine-pyrimethamine (n = 91)

Figure 2 is a Kaplan–Meier plot (survival analysis) showing gametocyte clearance times in patients who were gametocytaemic at enrolment, after treatment with AL (n = 42) and with AS + SP group (n = 91). By day 7, after treatment the proportion with gametocytaemia was significantly lower in the AL group compared with the ASSP group [20/42 (47.6 %) versus 68/91 (74.7 %), Chi = 9.432, P = 0.002]. This higher gametocyte clearance rate was observed at the follow-up visits on days 14, 21 and 28. Among the patients who were negative for gametocytes at baseline, more patients in the AS + SP group developed gametocytaemia after treatment than in the AL group, 22/342 (6.4 %) versus 5/214 (2.3 %), respectively, P = 0.041, Fisher’s exact test.

## Discussion

The 28-day parasitological cure observed after first-line AS + SP therapy in the present study exceeded 95 % in all the sites after PCR-correction and also in the uncorrected analyses, meeting the WHO recommendation that cure rates for falciparum malaria should be at least 90 % and preferably >95 %. Detection of asexual parasites on day 3 is an early warning sign of slow clearance of parasites by artemisinin [18, 19]. In the present study, the complete clearance of asexual parasitaemia in all cases on day 3 after treatment with AS + SP was an indication that the artesunate component is still effective. Since the blood levels of the drugs in the patients were note measured, it cannot be ruled out that these two cases had lower blood levels of the drugs. The SP dose was developed for adults but in the main target group (children aged 2–5 years) the weight-adjusted dose produced blood concentrations of both components that are approximately half of those in adults [20]. This means that the standard dose may be sub-optimal in younger children. Although in the present study AS + SP shows high efficacy resulting in a high cure rate, there is still a need for close monitoring of the therapeutic efficacy of this ACT in Yemen. Resistance to the SP component drugs are easy to induce experimentally [21]. In Africa SP resistance developed soon after the drug was adopted as first-line treatment against falciparum malaria [22]. This has been attributed mainly to the long elimination half-life of the SP components, which make it much easier for resistance to develop to this drug compared to the more rapidly eliminated anti-malarials [23]. The pattern of use of anti-malarial drugs is believed to be a major factor in the emergence and spread of anti-malarial drug resistance [24]. Nevertheless, it is notable that despite high rates of self-medication, incomplete treatments and sub-standard drugs, the efficacy of AS + SP remains high in Yemen, as shown in the present results [11, 25, 26].

The findings of high efficacy of AS + SP against falciparum malaria are supported by the fact that at present there is no evidence that SP has lost its efficacy against falciparum malaria in Yemen. In 2005, in vivo and in vitro tests were conducted by Al-Kabsi et al. to determine the SP efficacy against *P. falciparum* isolates from 100 malaria patients in Tihamah, Yemen [27]. In the in vivo test, no clinical or parasitological failure occurred. The in vitro test results suggested that SP is still effective against *P. falciparum* in the study area.

Published studies with SP resistance-associated molecular markers in Yemen are few and have not provided consistent results about the prevalence of these markers. A molecular marker study in Meseimeer found a 5 % prevalence of *dhfr* Arg-59 mutation in 99 amplified samples, while the *dhps* Glu-540 was not detected in 119 amplified samples [6, 7]. This was interpreted to suggest that the selection process had not reached *dhps* [6]. In a more recent work [28], four drug-resistant genes (*pfcrt*, *pfmdr1*, *dhfr,* and *dhps*) were genotyped in 108 *P. falciparum* isolates collected in three sites in Yemen: Dhamar, Hodeidah and Taiz. The investigators concluded that the absence of the triple mutant *dhfr* genotype (IRN) and *dhps* mutations supports the use of AS + SP as first-line therapy. They suggested that the previous report on the presence of C59 mutation could possibly be explained by inclusion of expatriates in the sample of patients studied. In another study, isolates from 90 patients with microscopically confirmed *P. falciparum* infection from Al-Hodaida were analysed for the molecular *dhfr* 108 N by Abdul-Ghani et al. [29]. The mutation was detected among about 61 % of *P. falciparum* isolates in its pure and mixed-type forms. They suggested that the high frequency of *dhfr* 108 N among parasite isolates should be cause for concern about the efficacy of SP as partner with AS. However, *dhfr* 108 mutation is a first step and is followed by other mutations before significant resistance occurs [30]. The high prevalence of SP resistance-associated genotypes reported from Sudan [31] and other countries in the region, such as Ethiopia [32] and Somalia [33], calls for close monitoring for the emergence of these genotypes in Yemen.

In the present study, the 28-day, PCR-uncorrected cure rate after treatment with AL is 100 % in Bajil (95 % CI 95.1–100 %), in Jabal-Al-Sharq it is 97.8 % (95 % CI 92.2–99.7 %) and in Tur Bani Qa’is it is 94.3 % (95 % CI 87.2–98.1 %). The PCR-corrected, 28-day cure rate after AL in each of the three sites is 100 %. This is higher than the findings of a recently pooled analysis of a 28-day, PCR-corrected parasitological cure rate of 97.1 % in adults and 97.3 % in children [34]. The two cases classified as LPF in Jabal-Al-Sharq study in the uncorrected analysis (PCR was not done) could be explained by low bio-availability of the drug but drug blood levels have not been done to confirm.

The effect of AL in the present study on microscopic gametocytaemia is consistent with findings from other endemic areas, which show a significant impact of AL on gametocytaemia [35]. The data also demonstrate the superior efficacy of AL over AS + SP in clearance of microscopic gametocytaemia. This could be a factor in choosing between different ACT in the future, particularly in an area of low transmission, on the path to malaria elimination.

## Conclusions

AS + SP remains a safe and effective first-line drug for the treatment of uncomplicated falciparum malaria in Yemen. However, monitoring the efficacy of this ACT should be continued since there is a high risk of failure of the SP component due to sub-therapeutic levels resulting from inadequate use of anti-malarial drugs in the country. Surveillance for SP resistance-associated molecular markers should also be monitored as a supportive tool to in vivo efficacy. AL is highly efficacious in Yemen and remains the appropriate option as a second-line treatment for uncomplicated falciparum malaria. AL shows higher efficacy than AS + SP in the clearance of microscopic gametocytaemia.

## Abbreviations

TET: therapeutic efficacy test
AL: artemether-lumefantrine
SP: sulfadoxine-pyrimethamine
ACT: artemisinin-based combination therapy
AS+SP: artesunate+sulfadoxine-pyrimethamine
AS: artesunate
S: sulfadoxine
P: pyrimethamine
ETF: early treatment failure
LCF: late clinical failure
LPF: late parasitological failure
ACPR: adequate clinical and parasitological response
LFU: lost to follow-up
WTH: withdrawal
CQ: chloroquine
CRF: case record form
WBC: white blood cell
PCR: polymerase chain reaction
